# Intestinal Fluid Permeability in Atlantic Salmon (*Salmo salar* L.) Is Affected by Dietary Protein Source

**DOI:** 10.1371/journal.pone.0167515

**Published:** 2016-12-01

**Authors:** Haibin Hu, Trond M. Kortner, Karina Gajardo, Elvis Chikwati, John Tinsley, Åshild Krogdahl

**Affiliations:** 1 Department of Basic Sciences and Aquatic Medicine, Faculty of Veterinary Medicine and Biosciences, Norwegian University of Life Sciences, Oslo, Norway; 2 Key Laboratory of Aquaculture Nutrition and Feed (Ministry of Agriculture) & Key Laboratory of Mariculture (Ministry of Education), Ocean University of China, Qingdao, Shandong, P. R. China; 3 BioMar Ltd., Grangemouth Docks, Grangemouth, United Kingdom; National Institute for Agronomic Research, FRANCE

## Abstract

In Atlantic salmon (*Salmo salar* L.), and also in other fish species, certain plant protein ingredients can increase fecal water content creating a diarrhea-like condition which may impair gut function and reduce fish growth. The present study aimed to strengthen understanding of the underlying mechanisms by observing effects of various alternative plant protein sources when replacing fish meal on expression of genes encoding proteins playing key roles in regulation of water transport across the mucosa of the distal intestine (DI). A 48-day feeding trial was conducted with five diets: A reference diet (FM) in which fish meal (72%) was the only protein source; Diet SBMWG with a mix of soybean meal (30%) and wheat gluten (22%); Diet SPCPM with a mix of soy protein concentrate (30%) and poultry meal (6%); Diet GMWG with guar meal (30%) and wheat gluten (14.5%); Diet PM with 58% poultry meal. Compared to fish fed the FM reference diet, fish fed the soybean meal containing diet (SBMWG) showed signs of enteritis in the DI, increased fecal water content of DI chyme and higher plasma osmolality. Altered DI expression of a battery of genes encoding aquaporins, ion transporters, tight junction and adherens junction proteins suggested reduced transcellular transport of water as well as a tightening of the junction barrier in fish fed the SBMWG diet, which may explain the observed higher fecal water content and plasma osmolality. DI structure was not altered for fish fed the other experimental diets but alterations in target gene expression and fecal water content were observed, indicating that alterations in water transport components may take place without clear effects on intestinal structure.

## Introduction

**Fig 1:**
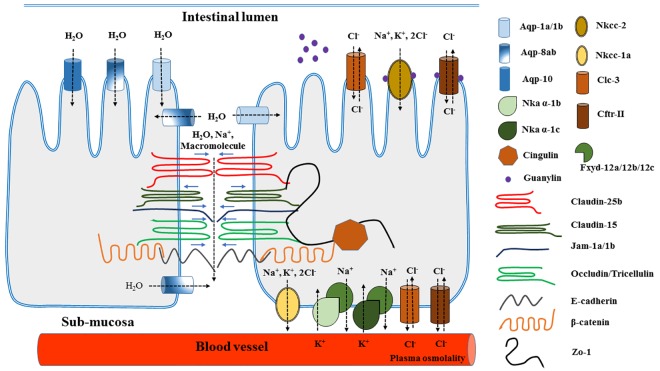
The elements involved in intestinal fluid permeability. Water is absorbed from the intestinal lumen into enterocytes through Aqp-1a/1b, Aqp-8ab and Aqp-10 located at the brush border and in sub-apical regions. Water is further drawn along the osmotic gradient out of enterocytes and into the sub-mucosa through basolaterally located Aqp-8ab and finally arrives in the blood vessels. The basolaterally located Nka α-1c or α-1b, regulated by Fxyd-12, are the main and primary activated elements in ion transcellular transportation and will transport Na^+^ out of enterocytes and K^+^ into enterocytes. The apically located Nkcc-2 contributes to absorption of Na^+^, K^+^ and Cl^-^ from the intestinal lumen and into enterocytes, whereas the basolaterally located Nkcc-1a secrets Na^+^, K^+^ and Cl^-^ from enterocytes into submucosa and finally into the blood vessels. The apically and basolaterally located Cftr-II and Clc-3 transport chloride ions into and out of enterocytes. Guanylin, secreted by goblet cells into the intestinal lumen could affect the function of apically located Nkcc-2 and Cftr-II. Claudin-25b, Jam-1a/1b, occludin, tricellulin, e-cadherin and β-catenin could tighten the junction barrier between enterocytes and limit the paracellular flux of water, ions and macromolecules, whereas claudin-15 is a pore-forming protein promoting paracellular flux of cations, predominantly Na^+^ and small molecules with radii <4Å. These junction proteins are linked to the actin cytoskeleton via binding to Zo-1 and cingulin. Black dotted arrows show the direction of water and ion transport, blue arrows show the function direction of junction barrier proteins. Abbreviations: Aqp, aquaporin; Nka, Na^+^, K^–^ –ATPase; Nkcc, Na^+^, K^+^, 2Cl^-^ co-transporter; Clc, chloride channel; Cftr, cystic fibrosis transmembrane conductance regulator Cl^-^ channel; Jam, junctional adhesion molecule; Zo, zonula occludens.

Feeding Atlantic salmon (*Salmo salar* L.) with diets containing high levels of alternative protein sources, especially soybean meal and certain other legumes, may induce digestive disturbances including diarrhea-like conditions indicating impaired gut permeability of water. Altered permeability may lead to impaired digestive functions and reduced fish growth [1–7]. Diarrhea-like phenomena are observed in particular in the distal compartment of the salmon gut. Similar observations have been made in rainbow trout (*Oncorhynchus mykiss*) [8–10]. Modulation of digesta water content by certain plant ingredients has also been observed in pigs [11]. The effect may be a result of variation in osmotic conditions as well as level and function of the many proteins involved in regulation of intestinal fluid permeability [8, 12–15]. The latter involves both transcellular and paracellular routes. Fig 1 illustrates the many elements involved in intestinal fluid permeability. Symptoms of diarrhea, when induced by legume ingredients in the diet are frequently accompanied by altered permeability also for other components [12]. Altered permeability may be the key mechanism in the often observed concomitant appearance of inflammation. Present knowledge on mechanisms underlying altered water permeability in diet-induced diarrhea in fish is very limited. The present work aimed to improve understanding of these mechanisms.

Incorporations of aquaporins (Aqp) and ion transporters in the intestinal epithelial membrane are key elements in regulation of animal intestinal transcellular fluid transport [13]. Previous studies have shown that the water channel proteins Aqp-8ab, Aqp-10, Aqp-1a (also named Aqp-1aa) and Aqp-1b (also named Aqp-1 or -1ab) are the main aquaporins in the Atlantic salmon intestine [13, 16]. Of these, Aqp-1a and Aqp-1b are located in the brush border and sub-apical region of enterocytes. Aqp-8ab is found in the same position as Aqp-1a/1b as well as in basolateral regions of enterocytes [13, 16]. The basolaterally located Na^+^, K^–^ –ATPase (Nka), the main Na^+^, Cl^-^ transporter, has been suggested as the most important and first activated element in regulation of ion-coupled transcellular fluid transport in the fish intestine [13, 17, 18]. Studies on salmon intestinal tissue have indicated that Nka α-1c and α-1b isoforms are the functional isoforms, with Fxyd-12 acting as a main modulating protein [18, 19]. In addition, Na^+^, K^–^, 2Cl^-^ co-transporters (Nkcc), especially the apically located absorptive isoform: Nkcc-2 and the basolaterally located secretory isoform: Nkcc-1a are playing key roles. The same seems to be the case for the chloride channels (Clc), such as cystic fibrosis transmembrane conductance regulator Cl^-^ channel II (Cftr-II) and Clc-3 in apical and basolateral membrane of enterocytes [18, 20]. Furthermore, guanylin, secreted by intestinal goblet cells into the gut lumen, seems to be correlated with diarrhea in fish intestine [6, 20, 21].

Paracellular permeability in the fish intestine appears to be regulated mainly via the network of tight junction proteins (TJP) and adherens junction proteins (AJP) between intestinal epithelial cells [13, 14]. Of tight junction (TJ) transmembrane proteins, claudins, especially claudin-15 and -25b, play key roles in intestinal fluid permeability in Atlantic salmon [13, 14, 19, 22]. Among these claudin-25b is barrier tightening whereas claudin-15 acts as a pore forming protein allowing paracellular flux of cations, predominantly Na^+^ and small molecules with radii <4Å. Other important barrier tightening TJPs are junctional adhesion molecule (Jam)-1, as well as occludin and tricellulin, which regulate water and large macromolecule flux presumably through the non-restrictive leak pathway [13, 14, 22]. These TJ transmembrane proteins bind to TJ cytoplasmic plaque proteins, mainly zonula occludens-1 (Zo-1), and then to cingulin [13, 14]. In addition, AJs, predominantly composed by cadherin-catenin interactions especially e-cadherins - β-catenin interaction, also regulate animal intestinal paracellular pathways [14].

The purpose of this study was to examine whether modulation of the various elements involved in water transport may explain the diarrhea-like symptoms observed in salmonids fed diets containing high content of alternative protein sources, i.e. soybean meal and soybean concentrate. Poultry meal and guar meal were also included in the present study as relevant alternative feed ingredients. Poultry meal is quite well described as a fish feed ingredient [23]. Guar meal, a byproduct of the guar gum industry containing 35–50% protein is less well known. No information has been found in the literature regarding its nutritional value and effects of guar meal on digestive physiology in fish. The presence of antinutrients such as trypsin inhibitors and saponins make the product interesting in comparison with soybean products among which some contain many antinutrients [24]. The following endpoints were selected to get a broad perspective on mechanisms that might be involved: expressions of genes coding for aquaporins, ion transporters, tight junction and adherens junction proteins in distal intestine, plasma osmolality, fecal dry matter and intestinal histology.

## Materials and Methods

### 2.1. Fish management

The experiment was conducted in strict compliance with laws regulating the experimentation with live animals in Denmark as overseen by the Danish Animal Experiments Inspectorate and since the present study was a feeding trial performed with Atlantic salmon, no further approval was required. The feeding trial was performed at BioMar’s RAS research facilities in Hirtshals, Denmark. For each diet, mixed-gender groups of 22 or 23 Atlantic salmon post smolt with an initial mean body weight of 314 g (SEM = 2 g) were randomly distributed into duplicate 0.8 m^3^ fiberglass tanks, containing 1000 L seawater replaced at a rate of 1600 L/min. The temperature was 15°C during the feeding trial. Oxygen saturation was above 85% throughout the duration of the trial, and salinity was maintained at 33 ± 1 g/L. Fish were fed continuously by automatic belt feeders 18 hours feeding period from 1 PM to 7 AM, and feed consumption was recorded daily. The uneaten pellets were removed and dried to estimate the feed intake. A regimen of 24 h lighting was employed during the experimental period.

### 2.2. Diets and feeding

Diets were produced at BioMar’s Tech Centre in Brande, Denmark with a pellet size of 4.5mm. Diet formulations and their chemical compositions are shown in Table 1. The control diet (FM) was formulated with fishmeal as the sole protein source. In the other four experimental diets fish meal was replaced with soybean meal mixed with wheat gluten (SBMWG), soy protein concentrate mixed with poultry meal (SPCPM), guar meal mixed with wheat gluten (GMWG) or poultry meal (PM). All diets were supplemented with vitamins and minerals to fulfill the fishes’ requirements for all nutrients. The diets varied somewhat in ingredient composition to be practical according to BioMar’s considerations. In the presentation and discussion of the results comparisons are therefore mainly based on the difference between the control diet and each of the other experimental diets, not the differences among the other diets. The fish were fed for 48 days.

**Table 1:** Formulation of the experimental diets^a^.

	FM	SBMWG	SPCPM	GMWG	PM
*Ingredients (g/kg)*					
Fish meal^b^	723.5	202.4	362.2	276.6	143.0
HiPro Soya		300.0			
Wheat gluten^c^		220.0		145.0	
SPC			300.0		
Guar meal				300.0	
Poultry meal^d^			60.0		580.0
Tapioca	110.0	110.0	110.0	110.0	110.0
Rape oil^e^	80.0	80.0	80.0	80.0	80.0
Fish oil^f^	80.0	80.0	80.0	80.0	80.0
Premix N^g^	6.0	7.1	7.3	7.9	6.5
Yttrium	0.5	0.5	0.5	0.5	0.5
*Chemical Composition (%)*					
Crude protein	51.4	47.0	46.4	49.9	49.4
Crude lipid	23.3	20.5	22.3	22.0	25.7
Starch	10.6	12.0	11.8	13.3	11.4
Ash	9.3	5.0	7.3	6.0	9.6
Phosphorus	1.4	0.7	1.0	0.8	1.6
Digestible energy (MJ/kg)	21.1	18.3	18.4	18.9	19.6

^a^ The diets were assigned abbreviations as indicated above the columns.

^b^ NA LT Fish meal (Norsild, NOR)

^c^ Wheat gluten (Lantmännen, SW)

^d^ Poultry meal (Gepro, D)

^e^ Rape oil (Emmelev-Scanola, DK)

^f^ Fish oil (FF Skagen, DK)

^g^ Premix N: BioMar premix”, footnote: minerals, vitamins and synthetic amino acids to cover the nutrient requirements of the species.

### 2.3. Sampling

At the termination of the experiment, the weight and length of all fish were recorded for calculation of growth performance. Samples were taken from six randomly selected fish from each duplicate tank. Samples were taken only from fish with digesta throughout the intestinal tract, to ensure intestinal exposure to the diets until sampling. All fish were fully anesthetized with benzocaine (20ml/100L) (Kalmagin 20%, Centrovet) and subsequently euthanized by cervical dislocation prior to tissue sampling. From all euthanized fish, blood was collected in heparinized vacutainers for plasma preparation, then frozen in liquid N_2_ and stored at −40°C for plasma osmolality measurement. The fish were manually stripped for feces by gently applying pressure to the lower abdominal region. Initial urine expression was removed and discarded before feces could be collected. Fecal samples were immediately frozen in liquid N_2_, freeze-dried and ground prior to analysis. Thereafter, the abdominal cavity was opened, and the intestine was removed and cleared of mesenteric and adipose tissue. Content from the distal intestine (DI) was collected quantitatively in separate, pre-weighed tubes, and then frozen in liquid N_2_ and stored at −80°C pending analyses. Distal intestinal tissue samples from the same six fish were collected for histological evaluation, placed in 4% phosphate-buffered formaldehyde solution for 24 h, and subsequently stored in 70% ethanol until further processing. For RNA extraction, DI tissue samples (~100 mg) from the same six fish were taken and placed in RNAlater (Ambion, Carlsbad, CA) at 4°C for 24 h, and were subsequently stored at −20°C.

### 2.4. Diet and feces analyses

Feed and feces samples were analyzed for dry matter (EU 71/393), Kjeldahl nitrogen (N) (EU 93/28), lipid (HCl hydrolysis and diethyl ether extraction (EU 98/64)), and starch (AOAC enzymatic method 996.11). Gross energy was measured by bomb calorimetry (Parr 1271 bomb calorimeter, Parr, Moline, IL). Amino acids (except tryptophan) analysis of all samples were performed with a Biochrom 30 amino acid analyzer (Biochrom Ltd., Cambridge, U.K.) after hydrolysis, according to EC Commission Directive 98/64/EC. Tryptophan was analyzed on a Dionex Summit HPLC system, with a Shimadzu RF-535 fluorescence detector. Yttrium oxide concentrations in feed and feces were determined by inductively coupled plasma mass spectroscopy (ICPMS) as previously described [25].

### 2.5. Histology

Histology slides with DI tissue sections from each fish stained with haematoxylin and eosin were evaluated using a semi-quantitative technique. All slides were evaluated using light microscopy. The evaluation procedure focused on the characteristic morphological changes of soybean meal induced DI enteritis in Atlantic salmon, that consist of changes in mucosal fold length, width and cellularity of the submucosa and lamina propria, enterocyte supranuclear vacuolization, and frequency of goblet cells, intra-epithelial lymphocytes, mitotic figures and apoptotic bodies within the epithelial layer. For each of the morphological characteristics, the degree of change was graded using a scoring system with a scale of 0–10 where 0–2 represented normal, >2 to 4 mild changes, >4 to 6 moderate changes, >6–8 marked changes, and >8–10 severe changes. Scoring was done using a visual analogue scale to generate the scores as continuous variables that allowed conducting one way analysis of variance (ANOVA) statistical analyses on the data.

### 2.6. Plasma osmolality

Blood plasma osmolality from each fish was determined by freezing point depression by a cryoscopic method using a Knauer osmometer (semi-micro-osmometer type ML. no. A0299, Berlin, Germany).

### 2.7. Quantitative Real Time PCR (qPCR)

Total RNA was extracted from DI tissue samples (~30 mg) from the first five fish in the first duplicate tank and the first four fish in the second duplicate tank per treatment by using Trizol reagent (Invitrogen™, Thermo Fisher Scientific, Waltham, MA, USA) and purified with Pure Link (Invitrogen™) including an on-column DNase treatment according to the manufacturer’s protocol. The integrity of the RNA samples was verified by the 2100 Bioanalyzer in combination with RNA Nano Chip (Agilent Technologies, Santa Clara, CA, USA). RNA integrity numbers (RIN) were >8 for all samples, with an average RIN of 8.9. RNA purity and concentrations were measured using the NanoDrop ND-1000 Spectrophotometer (Thermo Fisher Scientific). Total RNA was stored at −80°C until use. First-strand complementary DNA was synthesized from 0.8 μg total RNA from all samples using SuperScript® III First-Strand Synthesis SuperMix for qRT-PCR (Invitrogen™). Negative controls were performed in parallel by omitting RNA or enzyme. The qPCR primers for amplification of gene-specific PCR products were designed using Primer3web software version 4.0.0 (http://primer3.ut.ee/) or obtained from the literature. The primer details are shown in Table 2. All primer pairs were first used in gradient reactions in order to determine optimal annealing temperatures. To confirm amplification specificity, the PCR products from each primer pair were subjected to melting curve analysis and visual inspection of the PCR products by agarose gel electrophoresis. PCR efficiency for each gene assay was determined using 2-fold serial dilutions of randomly pooled complementary DNA. The expressions of individual gene targets (9 fish per group, 4 or 5 individuals from each tank duplicate) were analyzed using the LightCycler 96 (Roche Diagnostics, Basel, Switzerland). Each 10 μl DNA amplification reaction contained 2 μl PCR grade water, 2 μl of 1:15 diluted complementary DNA template, 5 μl LightCycler 480 SYBR Green I Master (Roche Diagnostics) and 0.5 μl (10mM) of each forward and reverse primer.

**Table 2:** Details of primer pairs used for real-time PCR assays

	5’-3’ primer sequence						
Gene name	Forward	Reverse	Amplicon size (bp)	Annealing temperature (°C)	Primer efficiency	GenBank accession no.	Primer reference
*aqp-1a*	CTACCTTCCAGCTGGTCCTG	TGATACCGCAGCCTGTGTAG	141	62	2.05	BT046625	[16]
*aqp-1b*	CTGTGGGTCTGGGACATCTT	TAAGGGCTGCTGCTACACCT	153	62	2.00	NM_001140000.1	[16]
*aqp-8ab*	GGAGCTGCCATGTCAAAGAT	CGCCCCTAGCAATACTACCA	159	60	2.00	KC626879.1	[6]
*aqp-10*	GGTGTTGGTGATCGGAGTCT	CGCCCTAAACACCTCATCC	121	60	1.68	DW569041	[16]
*nka α-1c*	GAGAGGGAGACGTACTACTAGAAAGCA	CAGCAAGACAACCATGCAAGA	69	60	2.01	NM_001124459	[26]
*nka α-1b*	CTGCTACATCTCAACCAACAACATT	CACCATCACAGTGTTCATTGGAT	81	60	2.00	NM_001124460	[26]
*fxyd-12a*	ATCCCTCCGTCATTGGTCAA	TGCTTCATAACTGCTTTCCCTG	96	62	2.01	NM_001123730.1	
*fxyd-12b*	AGGGCAGCTGACTACAGAGA	TGCATCAGGGTCGTACTCAG	139	55	1.96	BK006250	
*fxyd-12c*	GACCTTCGTTGCAGTCATCA	GGCTTCTCTTCAACCTTTCTTTC	102	62	2.00	BK006251	
*nkcc-1a*	GATGATCTGCGGCCATGTTC	CTCGTTCTTCATCAGCCAGC	100	60	1.97	NM_001123683.1	
*nkcc-2*	CCGCGTGCCCAACATC	GCACGGTTACCGCTCACACT	57	60	2.03	NM_001141520	[18]
*clc-3*	CGAGGGCATCTACGAATCACA	CTCCTTGGCGTCGAGGAA	61	60	1.95	NM _001173586	[18]
*cftr-II*	AGAATGGGACCGAGAAGTGG	CTCCCCAAAGTACAGCAGGA	115	62	1.91	NM_001123534.1	
*guanylin*	ATCTGGTCTCTGAAGCCCAC	CCGTTTACACTGCTGCTCTC	115	60	2.02	BT047912.2	
*claudin-15*	GGCACGTCTGAGAAACAACA	TAGGAAGTGGCAGCCTGACT	92	60	2.02	BK006395	[19]
*claudin-25b*	CCTGTAAGAGGGGTCCATCA	TGACACATGTTCTGCCCTGT	101	60	1.98	BK006399	[19]
*occludin*	GACAGTGAGTTCCCCACCAT	ATCTCTCCCTGCAGGTCCTT	101	60	2.12	NM_001173656.1	[22]
*tricellulin*	GGATGCCATGATGGGTAAAC	AGGAAGGCTGGGTCACTCTT	111	60	2.03	DW548339	[22]
*jam-1a*	TCGGCAGGCTATTCAAGAGT	AGTAGGTTCCTCTGGCACTG	110	60	2.00	GBRB01043957.1	
*jam-1b*	CGTTGCGGAAGGGCGTAG	CCAGCGATGTGTCCGATTTC	146	60	2.00	GBRB01043958.1	
*zo-1*	CAAAGCCAGTGTATGCCCAG	CAGCTTCATACTCGGCCTGA	119	60	1.93	>gnl|SRA|SRR403078.506834.2	
*cingulin*	AAAAGCACCCTGGAGAGACA	TTTGTTCCTCTCCTCCACCC	147	60	2.02	JT824570	
*e-cadherin*	ACTATGACGAGGAGGGAGGT	TGGAGCGATGTCATTACGGA	107	60	1.98	BT058864.1	
***β****-catenin*	ATGAGGACGCAGATGACCAA	CTGGGGTGTCTGGGAACTTT	126	62	2.07	NM_001140567.1	
*gapdh*	AAGTGAAGCAGGAGGGTGGAA	CAGCCTCACCCCATTTGATG	96	60	1.85	BT050045	[27]
*rnapoii*	CCAATACATGACCAAATATGAAAGG	ATGATGATGGGGATCTTCCTGC	157	60	1.80	BG936649	[27]
*hprt1*	CCGCCTCAAGAGCTACTGTAAT	GTCTGGAACCTCAAACCCTATG	255	60	1.99	BT043501	[27]

Abbreviations: *aqp*, aquaporin; *nka*, Na^+^, K^–^ –ATPase; *nkcc*, Na^+^, K^–^, 2Cl^-^ co-transporter; *clc*, chloride channel; *cftr-II*, cystic fibrosis transmembrane conductance regulator Cl^–^ channel II; *jam*, junctional adhesion molecules; *zo*, zonula occludens; *gapdh*, glyceraldehyde-3-phosphate dehydrogenase; *rnapoii*, RNA polymerase 2; *hprt1*, hypoxanthine phosphoribosyltransferase 1; *fxyd-12*, fxyd domain-containing ion transport regulator 12.

Each sample was assayed in duplicate, including a no-template control. The three-step qPCR run included an enzyme activation step at 95°C (5 min), forty to forty-five cycles at 95°C (10 s), 55–62°C (depending on the primers used, 10 s; see Table 2) and 72°C (15 s) and a melting curve step. Distal intestinal gene expression was normalized to the geometric average of glyceraldehyde-3-phosphate dehydrogenase (*gapdh*), RNA polymerase 2 (*rnapolii*) and hypoxanthine phosphoribosyltransferase 1 (*hprt1*) expression as evaluated elsewhere [27]. Mean normalized expression of the target genes was calculated from raw Cq values by relative quantification [28].

### 2.8. Calculation

The growth performances including specific growth rate (SGR), feed conversion ratio (FCR) and feed intake (FI), and apparent digestibility (AD) were calculated according to the previous studies [29, 30].

### 2.9. Statistical analysis

Data were tested for normality and variance homogeneity using the Shapiro−Wilk *W* goodness of fit test and the Bartlett test, respectively. When necessary, data were Log-transformed to achieve normal distribution (indicated by a superscript “†”). These data were subjected to one-way ANOVA using SPSS 20.0.0. Tank means were used as statistical units. When overall differences were significant (*P*<0.05), Tukey’s test was used to compare the means among individual treatments. Since some qPCR data did not fulfill the requirement of normal distribution after Log-transformation (indicated by a superscript “§”), the analysis was performed using the Wilcoxon/Kruskal−Wallis test followed by the post-hoc Steel-Dwass method to compare the means. Statistical analysis of these qPCR data were performed using JMP statistical software (version 10, SAS Institute, United States). The level of significance for all analyses was set at *P*<0.05. Mean values with different superscript letters within a column or a line indicated significant differences between groups (*P*<0.05). Two-tailed Pearson correlation coefficients were calculated to determine possible relations between plasma osmolality and intestinal gene expressions.

## Results

### 3.1. General fish performance and nutrient digestibilities

Neither SGR nor FI were significantly affected by replacement of the fish meal with any of the alternative protein sources. However, for all replacement diets, a significantly higher FCR was observed than that of the FM diet. The SBMWG diet increased ADs of protein and the amino acids Trp, Ser, Glu, Pro, Gly and Cys, whereas ADs of starch and energy decreased compared to the FM diet (Tables 3 and 4; Data shown only for essential amino acids and Cys). Lipid digestibility was not significantly affected by SBMWG diet. Fish in SPCPM group showed significantly decreased ADs of protein and the amino acids Val, Thr, Ile, Leu, Lys, Met, Ser, Glu, Pro, Gly, Ala and Asp (Tables 3 and 4). Also AD of energy in SPCPM treatment was significantly decreased compared to fish in the FM treatment. Fish fed the GMWG diet showed significantly decreased ADs of starch, energy, and the amino acids Val, Arg, Thr, Ile, Leu, Phe, Lys, Ala, Asp and Tyr (Tables 3 and 4), and the same trend was seen for AD of lipid, while significantly increased AD of amino acid Cys. The diet with PM reduced ADs of crude protein, all amino acids as well as energy (Tables 3 and 4), and the same trend was seen for AD of lipid.

**Table 3:** Fish growth performance, chyme water content and macronutrients apparent digestibility, and plasma osmolality

	SGR (%/day)	FI (%/day)	FCR^†^	Dig. Lipid %	Dig. Protein %	Dig. Starch %	Dig. Energy %	Chyme water %	Plasma osmolality mOsmol/kg
*One-way analysis of variance (ANOVA)*									
*P* value	0.06	0.30	<0.001	0.02	<0.001	<0.001	<0.001	<0.001	<0.01
Pooled SEM	0.04	0.02	0.02	0.5	1.2	1.9	0.8	0.45	1.3
*Mean values*									
FM	1.43	1.16	0.82^c^	97.5^ab^	89.3^b^	85.5^a^	91.0^a^	86.69^b^	295^b^
SBMWG	1.20	1.20	1.00^a^	98.7^a^	92.7^a^	70.8^bc^	86.3^b^	89.15^a^	305^a^
SPCPM	1.37	1.28	0.93^b^	97.8^ab^	86.9^c^	82.6^a^	84.4^bc^	88.58^a^	302^ab^
GMWG	1.28	1.24	0.96^ab^	95.5^ab^	88.4^bc^	66.0^c^	83.0^c^	86.38^b^	300^ab^
PM	1.15	1.14	0.99^ab^	95.1^b^	77.9^d^	78.4^ab^	82.3^c^	83.64^c^	293^b^

Abbreviations: SGR, specific growth rate; FCR, feed conversion ratio; FI, feed intake. For explanation of diet abbreviations see Table 1. Log-transformed data are indicated by †. Mean values with different superscript letters within a column are significantly different (*P* < 0.05).

**Table 4:** Apparent digestibility (%) of essential amino acids and Cys

	Val	Arg	Thr	Ile	Leu	Phe	Lys	His	Trp	Met	Cys
	*One-way analysis of variance (ANOVA)*										
*P* value	<0.001	<0.001	<0.001	<0.001	<0.001	<0.001	<0.001	<0.001	<0.001	<0.001	<0.001
Pooled SEM	1.3	1.1	1.4	1.2	1.3	1.1	1.0	1.3	1.7	1.2	2.2
*Mean values*											
FM	91.8^a^	94.7^ab^	91.1^a^	92.2^a^	93.5^a^	93.7^ab^	94.4^a^	91.7^ab^	86.9^b^	93.4^a^	79.8^b^
SBMWG	93.9^a^	96.1^a^	91.8^a^	94.3^a^	94.8^a^	95.9^a^	94.5^a^	93.6^a^	91.6^a^	95.0^a^	88.1^a^
SPCPM	88.7^b^	93.4^bc^	86.5^b^	89.7^b^	90.2^b^	91.3^bc^	92.4^b^	89.5^b^	86.1^b^	90.4^b^	74.5^b^
GMWG	88.5^b^	92.1^c^	87.2^b^	89.0^b^	89.1^b^	90.6^c^	92.4^b^	89.8^b^	87.1^b^	93.0^ab^	86.3^a^
PM	77.5^c^	82.3^d^	75.2^c^	79.0^c^	79.0^c^	82.6^d^	82.5^c^	78.2^c^	70.3^c^	81.1^c^	62.7^c^

For explanation of diet abbreviations see Table 1. Mean values with different superscript letters within a column are significantly different (*P* < 0.05)

### 3.2. Chyme water content, plasma osmolality and distal intestinal morphology

**Fig 2:**
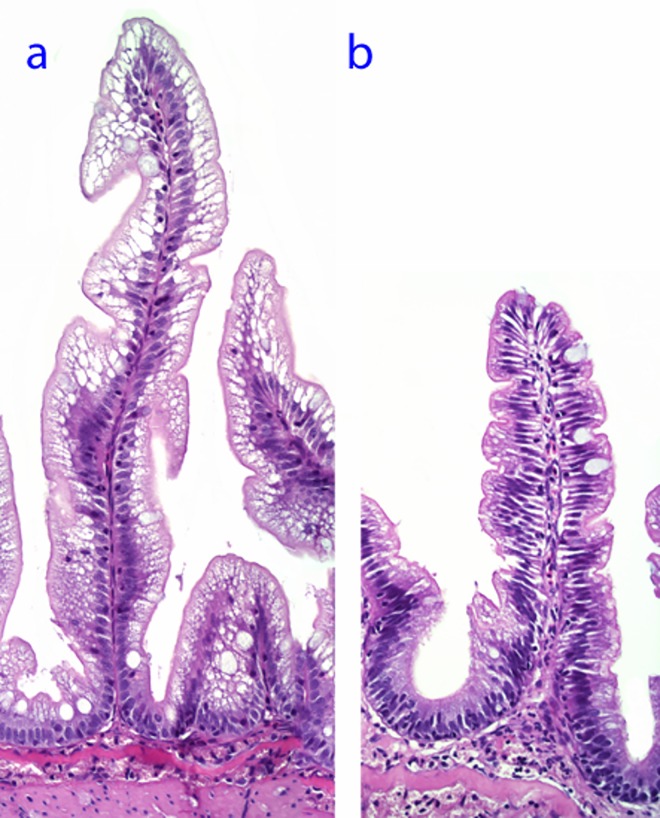
Representative histological appearances of distal intestine tissue scored as normal (a) and moderately altered (b)

**Fig 3:**
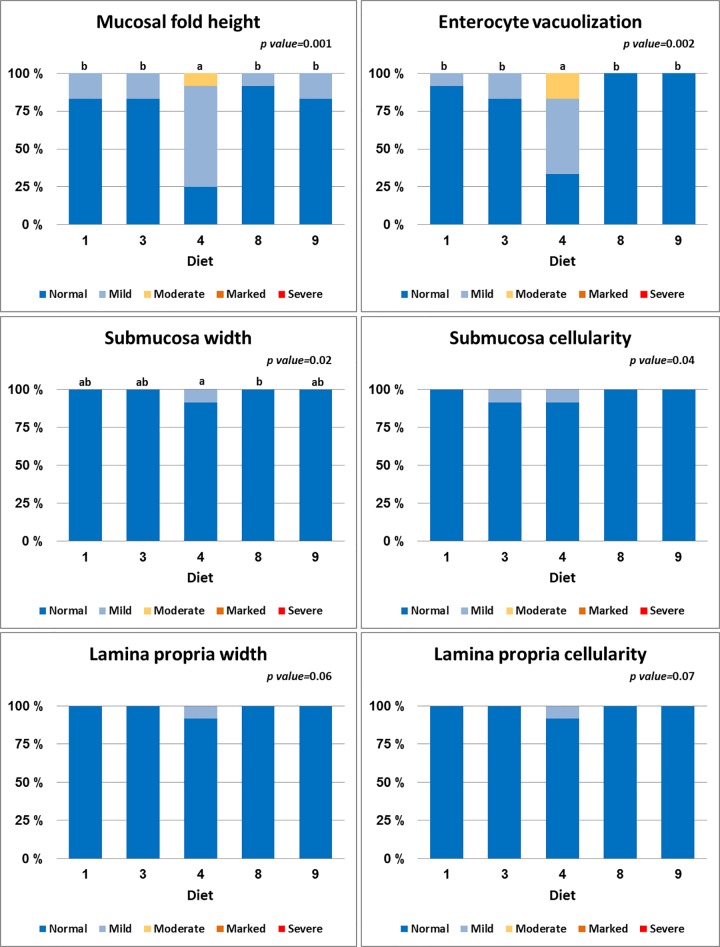
Contingency charts of the distal intestine morphology results. For explanation of diet abbreviations see Table 1. Contingency charts showing proportions of sampled individuals that scored “normal”, “mild”, or “moderate” (none scored above moderate) for selected distal intestine morphological characteristics. P values for the one-way ANOVA analyses are given.

Among the diets, SBMWG and SPCPM diets significantly increased chyme water content in the distal intestine (Table 3). The SBMWG diet also elevated plasma osmolality significantly whereas fish fed the SPCPM showed a clear trend in the same direction (Table 3). Alteration of DI morphology was seen only for the SBMWG diet with significant reductions in mucosal fold height and supranuclear vacuolization (Figs 2 and 3). As compared to the FM control diet, the GMWG diet did not cause clear effects either on chyme water content or plasma osmolality, whereas the PM diet decreased DI chyme water content without clearly affecting plasma osmolality (Table 3). Neither of the two latter diets altered DI histology.

### 3.3 Aquaporins, ion transporters and regulatory proteins in mucosa of the distal intestine

Fish fed the SBMWG diet showed the strongest effects on the observed gene expressions i.e. on aquaporins, ion transporters and their regulating proteins (Tables 5 and 6). Significantly lower expression of *aqp-8ab* and *aqp-10* was observed, and a similar trend could be seen for *aqp-1a* and *aqp-1b*. Regarding the apically located ion transporters *nkcc-2* and cystic fibrosis transmembrane conductance regulator Cl^-^ channel II (*cftr-II*) gene expressions were not significantly affected, but expression of the gene coding for guanylin, a regulator of the Nkcc and Cftr transporters, was significantly reduced. Among the observed genes coding for laterally located transporters, *nka α-1c* showed significantly lower expression. Significantly lower gene expression was observed also for the protein fxyd-12c, serving as a regulatory subunit of Nka and/or modulator of ion transporters. Expression of the other investigated ion transporters and modulators, *nka α-1b*, *nkcc-1a*, chloride channels (*clc-3*), *fxyd-12a* and *fxyd-12b* were not significantly altered by SBMWG feeding. Similar directions of alterations as seen in fish fed SBMWG diet were observed in fish fed SPCPM diet, but the effects were not significant except for the effect on expression of the *fxyd-12c*. Fish fed the GMWG diet showed significant reduction in *aqp-8ab* and *aqp-10*. In these fish neither *nkcc-2*, *cftr-II* nor *guanylin* expression showed significant effect, whereas *nka α-1c*, *fxyd-12a*, *fxyd-12b* as well as *fxyd-12c* showed significantly reduced expression. Fish fed the PM diet did not show significant alteration in expression of any of the aquaporins, ion transporters or their regulatory proteins as compared to fish fed the FM control diet.

**Table 5:** Gene expression levels of aquaporins in the distal intestine.

	*aqp-1a*^†^	*aqp-1b*^†^	*aqp-8ab*	*aqp-10*
*One-way analysis of variance (ANOVA)*				
*P* value	0.33	0.11	<0.001	0.001
Pooled SEM	0.002	0.0003	0.07	0.008
*Mean values*				
FM	0.013	0.0025	1.6^a^	0.20^a^
SBMWG	0.012	0.0021	0.7^b^	0.11^b^
SPCPM	0.015	0.0029	1.2^ab^	0.15^ab^
GMWG	0.013	0.0038	0.8^b^	0.12^b^
PM	0.022	0.0031	1.4^a^	0.17^ab^

For explanation of diet and gene abbreviations see Tables 1 and 2. Log-transformed data are indicated by †. Mean values with different superscript letter within a column are significantly different (*P* < 0.05).

**Table 6:** Gene expression levels of ion transporters in the distal intestine.

	*nkcc-2*^†^	*cftr-II*	guanylin^†^	*Nka α-1c*	*nka α-1b*^†^	*fxyd-12a*^†^	*fxyd-12b*^§^	*fxyd-12c*^§^	*nkcc-1a*	*clc-3*^†^
*One-way analysis of variance (ANOVA)*										
*P* value	0.44	0.77	0.03	<0.01	0.44	<0.001	0.02	<0.001	0.22	0.43
Pooled SEM	0.006	0.001	0.03	0.08	0.0003	0.003	0.005	0.13	0.0005	0.001
*Mean values*										
FM	0.09	0.026	0.69^a^	4.6^a^	0.0037	0.125^a^	0.174^a^	5.8^a^	0.0119	0.027
SBMWG	0.07	0.024	0.36^b^	4.0^b^	0.0047	0.108^ab^	0.159^ab^	4.2^c^	0.0102	0.034
SPCPM	0.05	0.024	0.54^ab^	4.2^ab^	0.0040	0.110^ab^	0.147^ab^	4.4^bc^	0.0103	0.029
GMWG	0.05	0.029	0.46^ab^	3.7^b^	0.005	0.094^b^	0.135^b^	4.3^c^	0.0109	0.027
PM	0.06	0.026	0.52^ab^	4.3^ab^	0.0045	0.131^a^	0.172^ab^	5.2^ab^	0.0134	0.028

For explanation of diet and gene abbreviations see Tables 1 and 2. The † indicates log-transformed data, § indicates data analyzed by Wilcoxon/Kruskal−Wallis test and post-hoc Steel-Dwass method. Mean values with different superscript letters within a column are significantly different (*P* < 0.05).

### 3.4 Tight junction and adherens junction proteins in mucosa of the distal intestine

Also regarding gene expression of the tight junction proteins the strongest effects were seen in fish fed the SBMWG diet (Table 7). Compared to FM fed fish, significantly decreased expression was observed for *claudin-15* and *cingulin* whereas expression of *occludin*, *jam-1b*, *e-cadherin* and *β-catenin* were significantly elevated. The same direction of alteration was observed for fish fed the SPCPM diet, but none of these alterations were significant. Fish fed the GMWG diet showed significant down regulation of *claudin-15*, similar to fish fed the SBMWG diet. For the other tight junction endpoints, changes in gene expression were not significant. In fish fed the PM diet, one significant effect was observed: elevation of expression of *occludin*.

**Table 7:** Gene expression levels of junction proteins in the distal intestine.

	*claudin-15*^†^	*claudin-25b*^§^	*occludin*^†^	*tricellulin*^†^	*jam-1a*^§^	*jam-1b*	*zo-1*	*cingulin*^†^		*e-cadherin*^§^	*β-catenin*
*One-way analysis of variance (ANOVA)*											
*P* value	<0.01	0.04	<0.001	0.05	0.03	0.02	<0.01	0.001		<0.01	0.04
Pooled SEM	0.06	0.04	0.006	0.007	0.002	0.006	0.004	0.0007		0.073	0.002
*Mean values*											
FM	1.7^a^	0.59	0.06^b^	0.14^b^	0.113^ab^	0.15^b^	0.089^ab^		0.012^a^	1.940^bc^	0.052^b^
SBMWG	1.1^b^	0.55	0.11^a^	0.14^b^	0.125^a^	0.20^a^	0.066^b^		0.005^b^	2.746^a^	0.066^a^
SPCPM	1.4^ab^	0.67	0.07^b^	0.16^ab^	0.111^ab^	0.17^ab^	0.079^ab^		0.008^ab^	2.143^b^	0.060^ab^
GMWG	1.2^b^	0.58	0.10^ab^	0.16^ab^	0.104^b^	0.15^b^	0.080^ab^		0.007^ab^	1.939^c^	0.063^ab^
PM	1.6^ab^	0.94	0.13^a^	0.20^a^	0.111^ab^	0.17^ab^	0.107^a^		0.011^a^	2.156^b^	0.053^b^

For explanation of diet and gene abbreviations see Tables 1 and 2. The † indicates log-transformed data, § indicates data analyzed by the Wilcoxon/Kruskal−Wallis test and post-hoc Steel-Dwass method. Mean values with different superscript letters within a column are significantly different (*P* < 0.05).

### 3.5. Correlation between plasma osmolality and gene expression

The mRNA levels of *aqp-1a*, *nka a-1c*, *fxyd-12a*, *fxyd-12b*, *fxyd -12c*, *nkcc-2*, *claudin-15* and *cingulin* in Atlantic salmon DI showed significantly negative correlations with fish plasma osmolality (*P*<0.05, Table 8).

**Table 8:** Two-tailed Pearson correlation coefficients between plasma osmolality and intestinal gene expressions.

	*aqp-1a*	*nka α-1c*	*fxyd-12a*	*fxyd-12b*	*fxyd-12c*	*nkcc-2*	*claudin-15*	*cingulin*
*r*	-0.30	-0.39	-0.38	-0.32	-0.43	-0.39	-0.37	-0.33
*P* value	<0.05	<0.05	<0.05	<0.05	<0.01	<0.01	<0.05	<0.05

For explanation of gene abbreviations see Table 2. Correlation was considered significant when *P*<0.05. Only significantly correlated genes are shown.

## Discussion

The results observed in fish fed the SBMWG diet gave the clearest basis for answering of the question of the present work: what are the physiological alterations underlying the elevated water content of chyme of the distal intestine? As the wheat gluten in the SBMWG diet has no identified antinutrients and has been found to have high nutritional value in Atlantic salmon, the causative agent was, in all likelihood, a soybean component [5, 31]. The observed effect of the SBMWG diet on chyme water content is in line with the results of numerous studies involving standard soybean meals, which when included in salmonid feed induce diarrhea-like conditions as well as enteritis [2–7]. The morphological alterations seen in the present study were less severe than often observed in Atlantic salmon fed diets with 30% soybean meal. The explanation may be related to the level of antinutrients in the specific soybean variety used, processing conditions of the product, ingredients of the diets interacting with the antinutrient effects, feed intake and/or genetic characteristics of the fish.

The results indicate that the elevation in chyme water content in fish fed the SBMWG was a result of reduced transport of water both into and out of the enterocytes via the aquaporins and ion transporters on both the apical and the basolateral side of the cell, i.e. as indicated by the observed decrease in expression of the genes *aqp-8ab* and *aqp-10*, *nka α-1c*, and the Nka associated, modulating gene *fxyd-12c*. The effect of the decrease in expression of *guanylin* is unclear as its product is known as an inhibitor of Na^+^-K^+^-transporters in the intestinal mucosa and known to cause diarrhea, i.e. reduced expression would counteract the observed effects on the aquaporins and ion transporters [32]. However, guanylin has also been suggested to exert the opposite effect, to increase intestinal ion and ion-coupled fluid absorption in fish in seawater by mobilizing Nkcc-2 activity via stimulating Cl^-^ supply into intestinal lumen through apical membrane Cftr-like channels [20, 21]. The effects observed on expression of tight junction and adherens junction proteins, i.e. decrease for *claudin-15* expressions and increase for *occludin*, *jam-1b*, *e-cadherin* and *β-catenin* expressions, all are expected to tighten the junction barrier and limit water and ion paracellular permeability [13, 14, 19, 22]. The results of the present study complement results of previous studies investigating effects of diet-induced distal intestinal inflammation on TJ function and fluid permeability in salmon. Similar to the observations in the present work, aquaporins, in particular *aqp-8*, were observed to be markedly reduced during distal intestinal inflammation in salmon, whereas *occludin* was found to be induced [6, 29, 33, 34]. Additionally, gene expression was reduced for several barrier tightening TJPs, including *claudin* and *cadherin* isoforms [6, 33, 34]. The study by Grammes et al. [33], as the present study, also showed decreased expression of pore-forming protein *claudin-15*.

The saponins in the soybean meal were, most likely, the main trigger of the observed effects as they, in pure form, have been found to induce enteritis in Atlantic salmon [6, 29]. Also other soybean antinutrients may be involved in aggravating the symptoms as indicated in studies with other animal species and cell models. Dietary inclusion of soya fiber (10%) was found to increase *occludin* and/or *zo-1* expressions in ileum and colon of weaning piglets [35] and soya oligosaccharides (0.5%) also have similar effects on weaning piglets [36]. Moreover, phytoestrogens such as soya genistein have been observed to increase *occludin*, *zo-1*, *e-cadherin* and *β-catenin* mRNA and protein expressions in the *Apc*^*Min/+*^ mouse model of colorectal cancer and in Caco-2 cells [37, 38]. Also dietary phytate (4.4 g/kg) may decrease Nka activity as shown in the duodenum and jejunum of chickens [39, 40]. Dietary β-conglycinin (8%) in a fishmeal diet was found to decrease Nka activity in all intestinal segments of Jian carp [41], and β-conglycinin hydrolysates (0.5 g/L) could increase tight junction barrier formation in Caco-2 cells [42]. Accordingly, several of the antinutrients in soya, alone or in combination, may be of the triggers of effects of the SBMWG diet on expression of genes coding for proteins involved in intestine fluid permeability in the present study.

Salt water fish are constantly losing water to the external hyperosmotic seawater across their body surfaces and via the kidney and need to drink salt water to maintain body osmolality [43]. Any factor reducing water absorption from the intestine would be expected to affect body osmolality as seen in the present study in fish fed the SBMWG diet. The higher plasma osmolality might further trigger thirst and stimulate fish drinking, which may aggravate the condition leading to diarrhea [44].

The moderate effects of the SPCPM diet observed on the elements involved in water transport across the intestinal mucosa indicate that processing of soybean meal into soy protein concentrate removes most but not all the antinutrients involved in gut effects of soybean meal. These results are consistent with the previous studies on Atlantic salmon showing decreased chyme dry matter as well as disturbances of nutrient digestibility when diets with a high level of soybean concentrate were fed [8, 45, 46]. Intestinal paracellular fluid permeability alteration caused by soy protein concentrate in the diet has also been found in the mouse colon as indicated by increased expression of barrier-tightening TJPs *claudin-1* and *occludin* mRNA [47].

Among the antinutrients mentioned above as possible triggers of the observed effects, saponins, phytoestrogens and oligosaccharides are removed during the alcohol water extraction in the production of the SPC. The potentially antinutritional effects of β-conglycinin and other protein antinutrients such as protease inhibitors and lectins are inactivated to a large extent by the denaturating effects of the solvents involved in the processing. Left among the antinutrients are fiber and phytate [48]. These might be partial reasons why the SPCPM diet changed ion-transporter expression of *fxyd-12c* in this study, as discussed above. Fish fed the diets with the two soybean products, i.e. the SMBWG and SPCPM diet, both showed increased and very similar DI chyme water content. This indicates that the mechanisms underlying this response may occur without symptoms of enteritis and even if the effects on expression of the proteins involved in water transport are minor. The components which the two products have in common, such as fiber (non-starch polysaccharides) and phytic acid are the most likely causative agent(s).

In the present study the GMWG diet decreased *aqp-8ab*, *aqp-10*, *nka α-1c*, *fxyd-12a*, *fxyd-12b*, *fxyd-12c* as well as *claudin-15* gene expression supposedly due to antinutrients in the guar meal [23]. However, fish chyme water content was not affected, in contrast to the SBMWG diet, which showed similar changes in gene expression. The observed difference in effect of the SBMWG and GMWG diet on chyme water content may indicate that also other mucosal components involved in control of water permeability components may play a role for the resulting chyme water content.

The decrease in chyme water content observed in the fish fed the PM diet did not seem to be related to major alterations in water transport across the intestinal mucosa of the distal intestine as only expression of one of the observed water transport variables was significantly affected. Occludin which is considered as a tight junction tightening protein, was significantly increased, an effect which would be expected to reduce water transport and rather increase chyme water content. The decrease in chyme water content was most likely a result of the low lipid digestibility and a resulting higher lipid content.

## Conclusions

Increased water content in chyme of the distal intestine of Atlantic salmon with corresponding increase in plasma osmolality was observed in fish fed a diet containing standard soybean meal. These fish also showed alterations in expression of aquaporins, ion transporters, associated proteins, tight junction and adherens junction proteins, which can explain the chyme and plasma alterations. The work also indicated that alterations in the water transport components may take place without alterations in chyme water content and plasma osmolality.
